# Neuroinflammation in Autism Spectrum Disorders: Role of High Mobility Group Box 1 Protein

**DOI:** 10.22088/acadpub.BUMS.6.3.148

**Published:** 2017-09-26

**Authors:** Valeria Dipasquale, Maria Concetta Cutrupi, Laura Colavita, Sara Manti, Caterina Cuppari, Carmelo Salpietro

**Affiliations:** 1 *Department of Adult and Childhood Human Pathology, Unit of Pediatrics, University Hospital of Messina, Messina, Italy.*; 2 *Department of Pediatrics, Hospital Umberto I of Siracusa, Siracusa, Italy.*

**Keywords:** Autism spectrum disorder, high mobility group box-1, marker, neuroinflammation, immunity

## Abstract

The pathogenesis of autism spectrum disorder (ASD) likely involves genetic and environmental factors, impacting the complex neurodevelopmental and behavioral abnormalities of the disorder. Scientific research studies emerging within the past two decades suggest that immune dysfunction and inflammation have pathogenic influences through different mechanisms, all leading to both a chronic state of low grade inflammation, and alterations in the central nervous system and immune response, respectively. The high mobility group box-1 protein (HMGB1) is an inflammatory marker which has been shown to play a role in inducing and influencing neuroinflammation. Current evidences suggest a possible role in the multiple pathogenic mechanisms of ASD. The aim of this manuscript is to review the major hypothesis for ASD pathogenesis, with specific regards to the immunological ones, and to provide a comprehensive review of the current data about the association between HMGB1 and ASD. A systematic search has been carried out through Medline via Pubmed to identify all original articles published in English, on the basis of the following keywords: “HMGB1”, “autism”, “autism spectrum disorder”, “neuroinflammation”, and “child”.

Firstly described by Kanner in 1943, autism spectrum disorder (ASD) is a lifelong disorder of neural development that occurs within the first three years of life, and it is characterized by deficits of social communication and repetitive and stereotyped behaviors and interests (1-3). An increasing trend in prevalence and incidence of ASD in the general population is observed worldwide (4). According to the most recent data coming from the centers for disease control and prevention, 1 in 88 children is affected, with a prevalence that is 4-5 times higher in boys than in girls (5). In the United States, the disease prevalence has been estimated at 14.6 per 1000 children, and 1 in 68 in children aged 8 years (6).

The pathogenesis of ASD is complex and still controversial. ASD risk is associated with genetic factors (7), but studies carried on identical twins suggest that there are more environmental components than previously believed (8). Many environmental factors associated with the risk of ASD have been identified, and one candidate is the host inflammatory response (9). The aim of this manuscript is to review the major hypotheses for ASD pathogenesis, with specific regards to the immunological ones, and to provide a comprehensive review of the current data about the association between HMGB1 and ASD. A systematic search was carried out through Medline via Pubmed (https:// www.ncbi .nlm.nih. gov/ pubmed) to identify all original articles published in English, on the basis of the following keywords: “HMGB1”, “autism”, “autism spectrum disorder”, “neuroinflammation”, and “child”. 

## Imunological dysfunction and inflammation in autism spectrum disorders

Both innate and adaptive branches of the immune system can impact neural development, cognitive functions, and behavioral pattern. From fetal development to adulthood, the immune system and central nervous system (CNS) interact with each other, influencing both systemic immune response (peripheral immune system) and local CNS immune function (the so-called ‘neuro-immunity’) (10). During fetal development, the activation of the maternal immune system may lead to changes in neural development; this is an important risk factor for ASD. Various interrelated factors may cause dysregulation of the maternal immune system. A study conducted by Zerbo et al. (11) found that maternal infectious diseases diagnosed at a hospital admission, especially bacterial ones, were related to increased risk of ASD. Infections during pregnancy, such as rubella (12- 14) or influenza virus (15), can create an inflammatory immune environment and trigger the production of maternal cytokines and chemokines, which can not only affect directly the placenta but also may cross the placenta, and enter in the fetal compartment, exerting effects on the fetus development (10). These effects can also be achieved in the absence of active infection, via generalized inflammatory response or loss of immune regulation (16). Animal models of maternal immune activation (MIA) have been particularly significant in highlighting the importance of maternal immune regulation (16-20). In addition, several studies have demonstrated that up to about 10% of mothers with ASD children and only 0–2% of controls have humoral antibodies against fetal brain proteins (10, 21-23). These anti-brain auto-antibodies can likewise gain access to the developing fetal brain and bind to fetal proteins, thereby impairing the course of neurodevelopment.

Human leukocyte antigen (HLA) genes on chromosome 6 and killer-cell immunoglobulin-like receptor (KIR) genes on chromosome 19 are two large multigene complexes interacting to eliminate unwanted virally infected and malignant cells, and seem to be linked to the risk of developing ASD (24). Data suggest that HLA alleles and KIR activating genes/haplotypes are common variants in different autism populations (24-26). For instance, an activating cB01/tA01 KIR gene-content haplotype and the cognate ligand HLA-C1k that activates this haplotype are significantly increased in ASD, thus increasing natural killer (NK)-cell killing (27). ASD subjects and their mothers have less HLA-G-mediated immune tolerance during pregnancy (28). At the uterine maternal/fetal interface, maternal NK-cells express leukocyte-associated immunoglobulin-like receptor (LAIR) and KIR molecules; they interact with HLA-G non-classical I molecules expressed on trophoblast cells during pregnancy to suppress normal immune responses (28). Guerini et al. (29) have shown that ASD subjects have an increase in a 14 bp insertion, and lower levels of soluble HLA-G protein.

Although ASD is not a classical immune-mediated disorder, there is an increasing interest in investigating the role of the immune system and inflammation in the development and persistence of the complex neurological and behavioral abnormalities related to ASD (30-32). Literature data strongly suggest that individuals with ASD differ in immune profile and markers, especially in the pro-inflammatory ones, from healthy individuals or those without ASD. Abnormal interplay between innate and adaptive immunity and CNS in ASD, leading to chronic low-grade inflammation in the CNS, has been reported (33-36). Lower percentage of CD4 T lymphocytes, skewed CD4:CD8 T cell ratio (31) and, more recently, altered function of T regulatory cells and NK cells have been found in ASD patients (37-39). Additionally, aberrant expression of many pro-inflammatory cytokines and chemokines, such as inteleukin-1(IL-1), interleukin-6 (IL-6), interleukin-8 (IL-8) and interleukin (IL-12), as well as macrophage migration inhibitory factor (MIF) and platelet derived growth factor (PDGF) has been demonstrated in ASD patients in peripheral blood, cerebrospinal fluid or brain tissues (40), and gastrointestinal system (40-42). Nonetheless, the results are still inconclusive. Saresella et al. (43) suggested the existence of an “autism endophenotype” that expands immune aberrations to relatives who are seemingly unaffected by the core symptoms of ASD, but present autistic traits, including delayed verbal, cognitive, and motor development. They showed systemic immunologic dysfunction in ASD children, such as the augmentation of pro-inﬂammatory and interleukin-10–producing immune cells, the increase of CD8+ naïve T lymphocytes, and the reduction of CD8+ effector memory and CD4+ terminally differenti-ated; similar immune dysregulation was also observed in related, unaffected siblings of autistic children, but not in healthy control subjects. Other studies further support evidence of a disturbed immune system with altered cytokine levels in ASD children compared with related or unrelated siblings (44-46). Napolioni et al. (46) found no significant differences in plasma-cytokine levels between children with ASD and their related non-autistic siblings. However, a significant association of cytokine levels – especially IL-1- with the quanti-tative traits and the clinical subgroups of ASD was found, confirming the impact of immune alterations on the core symptoms of ASD. Further studies are warranted.

## High mobility group box-1 protein and autism spectrum disorder

Levels of many immune markers have been shown to be altered in ASD, revealing a trend toward pro-inflammatory immune activity (9,41).

Some studies have demonstrated an association between serum levels of high mobility group box-1 protein (HMGB1), an inflammatory cytokine-like marker, and ASD-related symptoms incidence, prevalence and severity. For this reason, it is believed that this protein can play a role in both CNS-dysfunction and non-CNS abnormalities and co-morbidities associated with ASD, such as gastrointestinal disorders. A summary of the original research studies investigating the association between HMGB1 and autism spectrum disorder is provided in Table 1. HMGB1 is a highly conserved, intranuclear non-histone protein widely expressed in cells of vertebrates. It has a potent pro-inflammatory activity and can be passively released from necrotic cells or actively secreted in response to pro-inflammatory stimuli, either infectious (including lipopolysaccharides) or non-infectious, such as the so- called damage-associated molecular patterns (DAMPs) released from damaged tissues (47,48). Inflammatory cytokines, such as tumor necrosis factor alpha (TNF-α), IL-1, IL-6 and, IL-8 act as intermediate (49). Regulation of HMGB1 secretion is crucial for HMGB-1 mediated inflammation control, and depends on various processes such as phosphorylation (50), acetylation, and methylation (51). Once secreted, HMGB1 exerts its pro-inflammatory activity by activating the innate immunity through signal transduction in toll-like receptors 2/4 (TLRs-2/4) (52), and the receptor for advanced glycation end- products (RAGE) (53). HMGB1 is able to cross the blood-brain barrier. The brain cells may therefore be exposed to HMGB1 released both in the brain and in the periphery, including the intestinal mucosa (47, 54, 55). Saresella et al. (56) showed that the inflammasome system is activated in ASD. They stimulated ASD subject cells with adenosine triphosphate (ATP) and found that mRNA for the proteins whose assembly results in the formation of active inflammasome complexes are up-regulated; caspase-1 and inflammasome- associated cytokines, such as IL-1β and IL-18 are produced and activated in these patients. These data could possibly explain the origin of the neuroinflammation suggested to be present in this disease (43). Due to the well-known role of HMGB-1 in inflammasome activation (57), it would be interesting to address specific studies on a possible involvement of HMGB1, as another DAMPs, in the inflammasome assembly leading to pro- inflammatory cytokine production responsible of ASD-associated inflammation. Increased HMGB1 serum levels have been observed in inflammatory and autoimmune disorders, including Huntington disease, Alzheimer's disease, Parkinson's disease and multiple sclerosis (47). The existing scientific evidence indicates a possible involvement of HMGB1 in the pathogenesis of ASD. In 2010, Emanuele et al. (58) firstly suggested that HMGB1 can play a role in ASD. They observed higher HMGB1 blood concentrations in a sample of young adults with ASD than in healthy age- and gender- matched controls (10.8± 2.6 ng/mL versus 5.6±2.5 ng/mL respectively, P<0.001). Similar results were reported by Russo (59) and Babinskà et al. (49), who found high plasma levels of HMGB1 in ASD young and adult patients, compared to neurotypical controls. Moreover, it was found that HMGB1 plasma levels were related with low plasma concentration of epidermal growth factor (EGF), that is involved in growth and differentiation of cells in the CNS (60) and gastrointestinal tract (61). HMGB1 levels was also found to be related with epidermal growth factor receptor (EGFR), significantly elevated in the blood of autistic children (62), and hepatocyte growth factor (HGF), significantly decreased in blood of ASD children (mean age of 10 years) with severe gastrointestinal manifestations (63).

**Table 1:** Overview of the original research studies investigating the role of HMGB1 in autism spectrum disorder.

**Authors**	**Title**	**Study population**		**HMGB1 in ASD patients**				
		**Patients**	**Controls**	**Serum levels**	**Significant association**			
					EGF	EGFR	HGF	Symptoms
Emanuele et al. (58)	“Increased serum levels of high mobility group box 1 protein in patients with autistic disorder”	22 young adults	28 young adults	↑	__	__	__	Social interaction (ADI-R social scores)
Russo (59)	***“Decreased epidermal growth factor (EGF) associated with HMGB1 and increased hyperactivity in children with autism”***	38 children	40 children	↑	↓	__	__	__
Russo (62)	“Increased epidermal growth factor receptor (EGFR) associated with hepatocyte growth factor (HGF) and symptom severity in children with autism spectrum disorders (ASDs)”	33 children	34 children	↑	__	↑	↓	__
Babinská et al. (49)	***“Increased plasma levels of the high mobility group box 1 protein (HMGB1) are associated with a higher score of gastrointestinal dysfunction in individuals with autism”***	31 children and adults	16 children and adults	↑	_	_	_	Gastro-intestinal (GI score)

HMGB1: High mobility box1; ASD: autism spectrum disorders; EGF: epidermal growth factor; EGFR: epidermal growth factor receptor; HGF: hepatocyte growth factor; ADI-R: autism diagnostic interview-revised.

Furthermore, HMGB1 seems to have influence on ASD-related symptoms incidence, prevalence and severity. An independent direct association has been found between HMGB1 blood levels and the domain A scores in the autism diagnostic interview-revised (ADI-R), which reflects social deficits. Accordingly, higher HMGB1 levels were associated with a worse social interaction, thus, suggesting that this molecule could be considered as a biomarker related with the social interactions in this neurodevelopmental disorder (58). Non CNS-abnormalities seem to be involved too. Babinskà et al. (49) enrolled 31 children and young adults (26 males and 5 females) with severe autism, aged 2-22 years (mean age 9.0± 5.6 years), and assessed 6 types of gastrointestinal symptoms (abdominal pain, flatulence and bloating, diarrhea, constipation, pain in defecation, and abundant feces) through a questionnaire, investigating about their type, frequency and severity within the last three months. A score of gastrointestinal disorders (GI score) was calculated. Gastrointestinal problems were registered in 96.8% of autistic patients, which was higher than controls (66.6%). GI score prevalence and severity were higher in autistic patients with serum HMGB1 levels> 11 ng/mL than in patients with lower serum levels (83.3% vs 38.9%, respectively). HMGB1 is secreted by human inflamed intestinal tissues and is abundantly found in the stools of pediatric patients with inflammatory bowel diseases (IBDs), so that HMGB1 has been proposed as a novel marker of intestinal mucosal inflammation. This dosage may be a non-invasive method for clinical evaluation of the intestinal infectious diseases severity (64). In ASD patients, it has been demonstrated that the intestinal flora is dysfunctional (65), and it is believed that it may support a chronic low-grade inflammation and increased permeability of the intestinal wall, allowing inflammatory mediators to enter the circulation and likely cross the blood-brain barrier, hence influencing the brain function, including behavior (66, 67).

Nowadays possible clinical targeting of HMGB1 is being explored. Treatment with inhibitors of HMGB1 activity has been proven efficacious in reducing the inflammatory activity in a broad range of preclinical disease models (68).

## Conclusion

In summary, increased HMGB1 levels have been found in the blood of young and adult patients with autism spectrum disorder. The literature survey carried out in our paper shows that only few studies have investigated HMGB1 serum levels in subjects with ASD, and that all of them have been performed in small samples. Further limitations are the lack of additional standardized verification of autism spectrum disorder diagnosis and the possible autistic symptoms in healthy controls, and the lack of data about the severity of the disorder. It should also be distinguished between children and adult with ASD, and particularly between Asperger syndrome and high functioning autism in adults. More larger-scale studies are needed to be performed to clarify the role of HMGB1 in the pathogenesis of autism spectrum disorder, potentially leading to novel disease markers and targeted therapeutics.
